# Prediction of chromosomal abnormalities in the screening of the first trimester of pregnancy using machine learning methods: a study protocol

**DOI:** 10.1186/s12978-024-01839-5

**Published:** 2024-07-03

**Authors:** Mahla Shaban, Sanaz Mollazadeh, Saeid Eslami, Fatemeh Tara, Samaneh Sharif, Fatemeh Erfanian Arghavanian

**Affiliations:** 1 Department of Midwifery, Research Student Committee, Mashhad University of Medical Sciences, Mashhad, Iran; 2 Nursing and Midwifery Care Research Center, Mashhad University of Medical Sciences, Mashhad, Iran; 3 Department of Medical Informatics, Faculty of Medicine, Mashhad University of Medical Sciences, Mashhad, Iran; 4 Department of Obstetrics and Gynecology, Faculty of Medicine, Mashhad University of Medical, Mashhad, Iran; 5 Department of Medical Informatics, Faculty of Medicine, Mashhad University of Medical Sciences, Mashhad, Iran

**Keywords:** Machine learning, Chromosomal abnormalities, Prediction, Pregnancy

## Abstract

**Background:**

For women in the first trimester, amniocentesis or chorionic villus sampling is recommended for screening. Machine learning has shown increased accuracy over time and finds numerous applications in enhancing decision-making, patient care, and service quality in nursing and midwifery. This study aims to develop an optimal learning model utilizing machine learning techniques, particularly neural networks, to predict chromosomal abnormalities and evaluate their predictive efficacy.

**Methods/ design:**

This cross-sectional study will be conducted in midwifery clinics in Mashhad, Iran in 2024. The data will be collected from 350 pregnant women in the high-risk group who underwent screening tests in the first trimester (between 11-14 weeks) of pregnancy. Information collected includes maternal age, BMI, smoking habits, history of trisomy 21 and other chromosomal disorders, CRL and NT levels, PAPP-A and B-HCG levels, presence of insulin-dependent diabetes, and whether the pregnancy resulted from IVF. The study follows up with the women during their clinic visits and tracks the results of amniocentesis. Sampling is based on Convenience Sampling, and data is gathered using a checklist of characteristics and screening/amniocentesis results. After preprocessing, feature extraction is conducted to identify and predict relevant features. The model is trained and evaluated using K-fold cross-validation.

**Discussion:**

There is a growing interest in utilizing artificial intelligence methods, like machine learning and deep learning, in nursing and midwifery. This underscores the critical necessity for nurses and midwives to be well-versed in artificial intelligence methods and their healthcare applications. It can be beneficial to develop a machine learning model, specifically focusing on neural networks, for predicting chromosomal abnormalities.

**Ethical code:**

IR.MUMS.NURSE.REC. 1402.134

## Abstract (plain language summary)

Approximately 3% of newborns are affected by congenital abnormalities and genetic diseases, leading to disability and death. Among live births, around 3000 cases of Down syndrome (trisomy 21) can be expected based on the country's birth rate. Pregnant women carrying fetuses with Down syndrome face an increased risk of pregnancy complications. Artificial intelligence methods, such as machine learning and deep learning, are being used in nursing and midwifery to improve decision-making, patient care, and research. Nurses need to actively participate in the development and implementation of AI-based decision support systems. Additionally, nurses and midwives should play a key role in evaluating the effectiveness of artificial intelligence-based technologies in professional practice.

## Background

Congenital abnormalities and genetic diseases lead to disability and death in approximately 3% of newborns [1]. Chromosomal disorders, including trisomy 21, trisomy 18, trisomy 13, and sex chromosome disorders, affect about 1 in 150 live births [2]. These disorders can lead to physical and psychological challenges in affected children and an increased risk of pregnancy complications for pregnant women [3–6]. The screening tests for aneuploidy involve assessing certain hormone levels and using ultrasound to measure nuchal translucency [7–9]. Screening in the first trimester of pregnancy includes two biochemical markers: human chorionic gonadotropin (βhCG Free) and plasma protein A concentration (PAPP-A), along with the measurement of nuchal translucency by ultrasound, which is performed between the 11th and 14th weeks of pregnancy [3]. High-risk individuals may undergo invasive procedures like amniocentesis or chorionic villus sampling. However, these procedures can have complications and may increase stress and anxiety levels among mothers [10–12]. Additionally, studies have shown that a small percentage of cases identified as high-risk actually have aneuploidy [1, 13]. Midwives play a crucial role in providing advice and care to mothers during pregnancy, delivery, and postpartum, offering emotional support to reduce anxiety and stress [14]. In recent times, there has been a surge of interest in artificial intelligence (AI) methods such as machine learning and deep learning worldwide. These methods are being integrated into nursing and midwifery to enhance decision-making, patient care, service delivery, and research studies. It is essential for nurses to be actively engaged in the development and implementation of AI-based decision support systems, particularly when these systems impact their direct patient care. Additionally, nurses and midwives should play a more active role in conducting detailed and interdisciplinary research to assess the clinical, ethical, and legal implications of AI-based technologies in professional practice [15–17]. Machine learning, a subset of computer science and AI, focuses on deploying data and algorithms to imitate human learning and steadily improve accuracy. This technique involves developing algorithms that can learn from experience to enhance system performance, using data as the source of experience to build predictive models [18–20]. Nurses and midwives should be actively involved in the development and implementation of AI-based decision support systems. Machine learning aims to create machines that can learn and make decisions without direct programming, and it can help predict chromosomal abnormalities, potentially aiding in decisions about procedures for pregnant mothers. The aim of the present study is to create a machine-learning model, focusing on neural networks, to predict chromosomal abnormalities.

### Main goal

Predicting chromosomal abnormalities during the first three months of pregnancy through machine-learning techniques.

### Specific objectives:

1. Assessing the sensitivity of the optimized neural network in predicting chromosomal abnormalities during the first-trimester screening.
2. Identifying the key characteristics of the optimized neural network for predicting chromosomal abnormalities during the first-trimester screening.
3. Contrasting the performance of the optimized neural network with decision trees in diagnosing chromosome abnormalities during the first-trimester screening.
4. Contrasting the performance of the optimized neural network with random forest in diagnosing chromosome abnormalities during the first-trimester screening.

## Research inquiries:

1. How sensitive are optimized neural networks in predicting chromosomal abnormalities during first-trimester screening?
2. What are the distinguishing features of an optimized neural network in predicting chromosomal abnormalities during first-trimester screening?
3. Is there a significant difference between the results of the optimized neural network and the decision tree in diagnosing chromosomal abnormalities during the first three months?
4. Do the optimized neural network results differ from random forest results in detecting chromosomal abnormalities during first-trimester screening?

## Methods/design

### Study design

In this study, a cross-sectional approach will be used. It involves data from 350 pregnant women who underwent the first-trimester screening test at 11 to 14 weeks of pregnancy at the Mashhad clinic and were classified as high-risk. After receiving approval from the Medical Ethics Committee of the University of Sciences Mashhad Medicine and a letter of recommendation from the faculty of Midwifery Nursing of Mashhad, the researcher contacted the research centers, obtained necessary permissions, and began sampling at midwifery clinics each day. Data collection entails assessing factors such as the mother's age, BMI, maternal smoking, trisomy 21 history, CRL level, NT, PAPP-A, B-HCG, presence of insulin-dependent diabetes, and IVF pregnancy status. This data is gathered during visits to obstetric clinics for first-trimester screening results, with follow-up amniocentesis for those deemed high-risk. The study aims to diagnose chromosomal abnormalities accurately using first-trimester screening parameters to reduce stress associated with unnecessary amniocentesis testing. Initially, the required data is collected with a designated sample size and undergoes pre-processing. This involves managing missing values, eliminating anomalies, and standardizing the data. Subsequently, the researcher conducts statistical analyses on all input characteristics in the first phase to unveil significant relationships with the response variable, specifically regarding chromosomal abnormalities 13, 18, and 21. Moving on to the second phase, a predictive model is constructed employing machine learning techniques. In this study, two model variants are developed using all input features and influential features for decision-making. While the first model utilizes data gathered from pregnant women in its entirety, the second model employs filter-based feature selection methods to pinpoint essential features for building a prediction model. The process of model creation encompasses training and evaluation stages where K-fold cross-validation is employed to gauge model efficiency and performance. The model's decisions are juxtaposed with actual patient data from the dataset to compute model error, aiming to minimize it. Furthermore, the study will focus on constructing a model based on Artificial Neural Networks (ANN), seeking to optimize the network's structure and parameters. Given the pivotal role of structure and hyperparameters in network performance and prediction accuracy, an optimization approach such as Particle Swarm Optimization (PSO) will be leveraged to pinpoint optimal hyperparameter values and network structure. This optimization process is envisaged to enhance the accuracy of the prediction model in identifying chromosomal abnormalities post the initial screening, alongside other methodologies. Additionally, machine learning techniques like decision trees will be utilized for comparative analysis of results.

### Sample size and sampling method

In machine learning methods, sample size is typically not fixed; the more data available, the more efficient to enhance model effectiveness. With a significance level of 0.07 confidence level of 99% (i.e., z = 2.58), and precision of 0.05, a minimum of 173 individuals were calculated using the formula. The final sample size was set at 190 individuals, accounting for a ten percent dropout rate. While this calculation is customary in statistical methods, for machine learning models, a larger dataset of at least 350 individuals is necessary for more precise model design and comprehensive evaluation.

$$\begin{aligned} z=2/58\quad \mathrm p=0/07\quad \mathrm d=0/05\\ \mathrm N=\frac{\mathrm z^2\times\mathrm p\times\left(1-\mathrm p\right)}{\mathrm d^2} \end{aligned}$$

### Inclusion criteria

First-trimester screening and NT ultrasound between 11-14 weeks of pregnancy, along with amniocentesis.

### Exclusion criteria

Mother's unwillingness to participate, presence of twins or multiples, failure to undergo amniocentesis for high-risk screening cases.

### Study implementation platform and data collection locations

Midwifery clinics in Mashhad hospitals served as the research setting.

### Recruitment approach

Researchers conducted sampling in selected centers, convenience sampling, and collected necessary data after obtaining participants' consent.

### Data analysis

To ensure a reliable and standardized assessment of the prediction model, we employ the K-fold cross-validation method. This approach gauges the model's ability to generalize to new data by partitioning the dataset into k subsets. Training and evaluation are conducted on these subsets, enhancing system reliability through the assessment of varied random batches. Subsequently, results for accuracy, precision, sensitivity, and specificity are provided to assess the model's predictive capacity effectively.

## Discussion

Aneuploidy screening tests are divided into three categories: first-trimester screening, second-trimester screening, and combined first and second-trimester screening. First-trimester screening involves evaluating human chorionic gonadotropin (βhCG Free) and plasma protein A concentration (PAPP-A) and measuring nuchal translucency using ultrasound between the 11th and 14th weeks of pregnancy [7–9]. After first-trimester screening, high-risk individuals are recommended to undergo amniocentesis or chorionic villus sampling. However, these invasive procedures are time-consuming and expensive. Studies show that common complications of amniocentesis include fetal death, bleeding, and amniotic fluid leakage, premature rupture of membranes, amnionitis, and spontaneous abortion [7, 21, 22]. Research also suggests that amniocentesis can lead to increased stress and anxiety levels among mother [14] .According to a study by Hassanzadeh et al., only 10% of high-risk cases identified through first-trimester screening were confirmed as aneuploidy by amniocentesis [1]. Additionally, a study by Delkhosh et al. found that 5.2% of cases suspected of trisomy 21 during first and second-trimester screenings through amniocentesis were found to have aneuploidy [13]. Midwives play a crucial role in advising and caring for mothers during pregnancy, delivery, and postpartum. They provide emotional support to reduce mothers' anxiety and stress, ensuring the health of both mother and fetus and making pregnancy safe**.** The role of Utilizing artificial intelligence methods, such as machine learning and deep learning, in nursing and midwifery to greatly improve decision-making, patient care, service delivery, and research studies can be significant. It is imperative that nurses and midwives actively engage in the development and implementation of AI-based decision support systems. Machine learning aims to create machines that can learn and make decisions without direct programming, and it has the potential to accurately predict chromosomal abnormalities, thereby playing a crucial role in decisions about procedures for pregnant mothers.

## Data Availability

No datasets were generated or analysed during the current study.
